# Co(II) Recovery from Hydrochloric Acid Solution Using Menthol-Based Deep Eutectic Solvents (DESs): Application to NMC Battery Recycling

**DOI:** 10.3390/molecules30224414

**Published:** 2025-11-14

**Authors:** María Isabel Martín-Hernández, María Lourdes Rodríguez, Irene García-Díaz, Gorka Barquero-Carmona, Lorena Alcaraz, Olga Rodríguez-Largo, Félix A. López

**Affiliations:** Centro Nacional de Investigaciones Metalúrgicas (CENIM-CSIC), Avda. Gregorio del Amo, 8, 28040 Madrid, Spain; lourdesrodriguezcea@cenim.csic.es (M.L.R.); irenegd@cenim.csic.es (I.G.-D.); gorkbark@cenim.csic.es (G.B.-C.); alcaraz@cenim.csic.es (L.A.); olga.rodriguez@csic.es (O.R.-L.)

**Keywords:** deep eutectic solvents, critical metals, black masses, lithium-ion batteries, Co(II) recovery, energy transition

## Abstract

Lithium-ion batteries are essential to ensure electric mobility and reduce CO_2_ emissions from transportation. One of the most commonly used chemistries is nickel–cobalt–manganese (NMC) batteries, which also have applications beyond the automotive sector. The recycling of these batteries requires the development of technologies to enable the selective separation and recovery of the metals present in the battery. One of these selective technologies involves the use of deep eutectic solvents (DESs). This research study investigates the different parameters that influence the recovery of Co(II) from hydrochloric acid medium using the deep eutectic solvent 3 Aliquat 336:7 L-Menthol. Firstly, using synthetic Co(II) solutions, the parameters influencing the cobalt extraction process are examined, and then these optimal conditions are applied to the recovery of cobalt from solutions obtained by dissolving NMC 622 battery black mass in 10 M HCl. The obtained results show that the DES used is highly selective for Co(II) recovery compared to other metals present in the solution (Ni, Li and Mn), achieving recoveries of up to 90% of the cobalt initially present in solution. Stripping with H_2_SO_4_ 0.5 M allows the recovery of cobalt as a crystalline monohydrate salt (CoSO_4_.H_2_O). The optimization of the Co/Cu separation conditions is carried out, achieving the separation of Cu(II) using Aliquat 336 in kerosene.

## 1. Introduction

The transition to a zero CO_2_ emissions economy is accelerating the development of new energy production technologies. In December 2019, the European Commission introduced the European Green Deal [1] to make Europe climate-neutral by 2050.

Critical raw materials (CRMs) are key materials for renewable energy technologies and electric mobility. Given the declining availability of essential raw materials [2,3,4,5] such as indium, tin, lithium, cobalt, magnesium, rare earths, copper, etc., and the difficulty of obtaining them, the proposal of the recycling and recovery of critical metals has arisen; however, most of these resources currently exhibit low recovery rates at the end of their useful lives. The consumption of CRMs is particularly relevant in the development of electric mobility, since the production of lithium-ion batteries requires the consumption of a significant amount of metals [6]. Therefore, it is essential to develop technologies for the recovery of the metals present in these batteries [7,8].

Various methods have been employed for metal recovery from waste and by-products. Pyrometallurgy is a commonly used technique, offering the retrieval of high-purity alloys. However, this energy-intensive approach involves high temperatures, and there is a risk of metal losses in slag, resulting in lower recovery rates compared to other methods [9]. On the other hand, hydrometallurgical recovery is also prevalent for metal separation in aqueous medium [10]. This method achieves higher metal recovery rates with lower energy consumption and enables the treatment of low-concentration aqueous solutions, yet further research is needed to mitigate the environmental impact associated with hydrometallurgical processes [11] due to their use of mineral acids in processes such as leaching or solvent extraction. Solvent extraction is a mature and widely used technology with numerous advantages, such as low operating costs. However, the organic solvents (typically hydrocarbons) and extraction agents (organophosphorus compounds, amines, etc.) used in large quantities contribute to the environmental impact of the process. The replacement of these mineral acids, organic solvents and extraction agents is a key aspect of improving the environmental impacts of hydrometallurgical processes, which would make these processes more selective and less harmful to the environment [12]. Deep eutectic solvents (DESs) have emerged as a new class of green designer solvents, which can contribute to the use of cleaner processes. They are derived from renewable raw materials, easily accessible, cost-effective, reusable, energy-efficient in synthesis, low in toxicity, biodegradable, and thermally stable [13,14]. Unlike conventional solvents (ethanol, methanol, hexane, etc.), DESs are non-volatile, which means they have a very low vapor pressure and are therefore hardly flammable. DES’s have become more widely used in the last 5 years as an alternative to pyro- and hydrometallurgical methods for the extraction of different metals such as lithium, cobalt, nickel, and manganese [15,16,17,18].

Deep eutectic solvents are derived from blending two or three substances with specific compositions, wherein the individual components possess higher melting points than the resulting mixture. This mixture comprises a well-matched combination of a hydrogen bond donor (HBD) and a hydrogen bond acceptor (HBA) [14]. The reduction in the melting point is attributed to the extensive intermolecular hydrogen bonding and the charge delocalization [19]. There are two types of DESs: hydrophilic and hydrophobic. Both types of DESs have been previously used for metal recovery in solution [20]. The hydrophilic DESs have been described as leaching agents [21,22], whereas hydrophobic DESs have been widely employed as extraction agents by creating biphasic systems with hydrophilic compounds like water, for instance [23,24]. Recently Xue K et al. [25] have studied lithium extraction from aqueous media using hydrophobic deep eutectic solvents. The hydrophobicity of DESs depends on the chemical nature of the eutectic mixture, i.e., the HBA and the HBD.

The hydrophobic DES obtained in this study from Aliquat 336 and L-Menthol has previously been utilized for the extraction of different metals such as Li, Co, Ni, Fe, etc [26,27]. However, despite the conducted studies, it is necessary to delve deeper into the understanding and the effect of the different parameters influencing the extraction, selective separation, and recovery of cobalt using the 3 Aliquat 336:7 L-menthol DES as the extraction agent. In the present work, this DES is employed to investigate and optimize the parameters influencing cobalt extraction, utilizing a synthetic Co(II) solution for this purpose, and subsequently, the optimal conditions are applied to the selective extraction and separation of Co(II) by solutions obtained from the hydrochloric acid leaching of six black mass samples (black mass is a black material obtained from the processing of used batteries after dismantling and crushing them) originating from NMC 622-type lithium-ion batteries. Therefore, L-Menthol-based DES will represent a breakthrough in cobalt extraction compared to other, less environmentally friendly processes, with the consequent advance that this represents compared to other processes used previously. Optimization of the Co/Cu separation conditions is carried out, achieving the separation of Cu(II) using Aliquat 336 in kerosene.

## 2. Results and Discussion

### 2.1. Characterization of DES

#### 2.1.1. Fourier-Transform Infrared Spectroscopy

**Figure 1:**
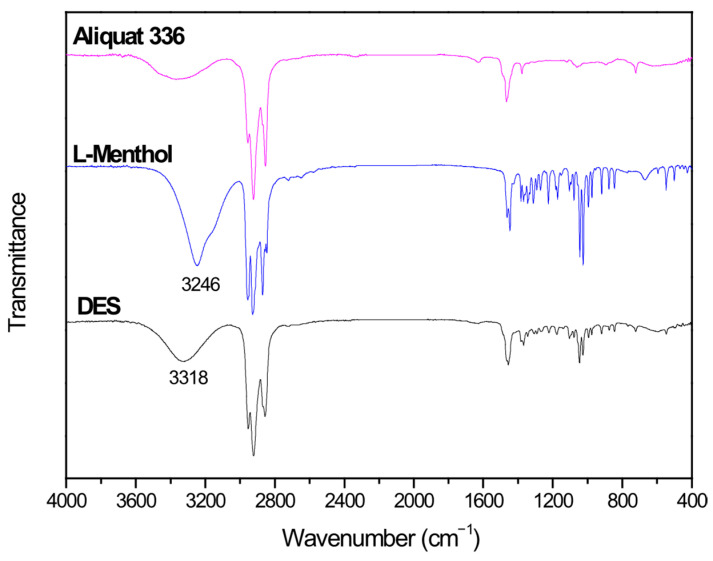
FTIR spectra of Aliquat 336, L-Menthol and deep eutectic solvent (DES) (3 Aliquat 336:7 L-Menthol).

DES is formed by hydrogen bonds between Aliquat 336 and L-Menthol. FTIR spectra of Aliquat 336, L-Menthol and DES are shown in Figure 1. This figure shows the presence of an OH vibration peak at 3246 cm^−1^ for L-Menthol. When DES is formed, the OH vibration of the DES is shifted to 3318 cm^−1^. This shift in OH vibration (72 cm^−1^) confirms the presence of hydrogen bonds between L-Menthol (HBD) and Aliquat 336 (HBA) when DES is obtained [27,28].

#### 2.1.2. Differential Scanning Calorimetry (DSC) and Thermogravimetry (TGA) of DES

**Figure 2:**
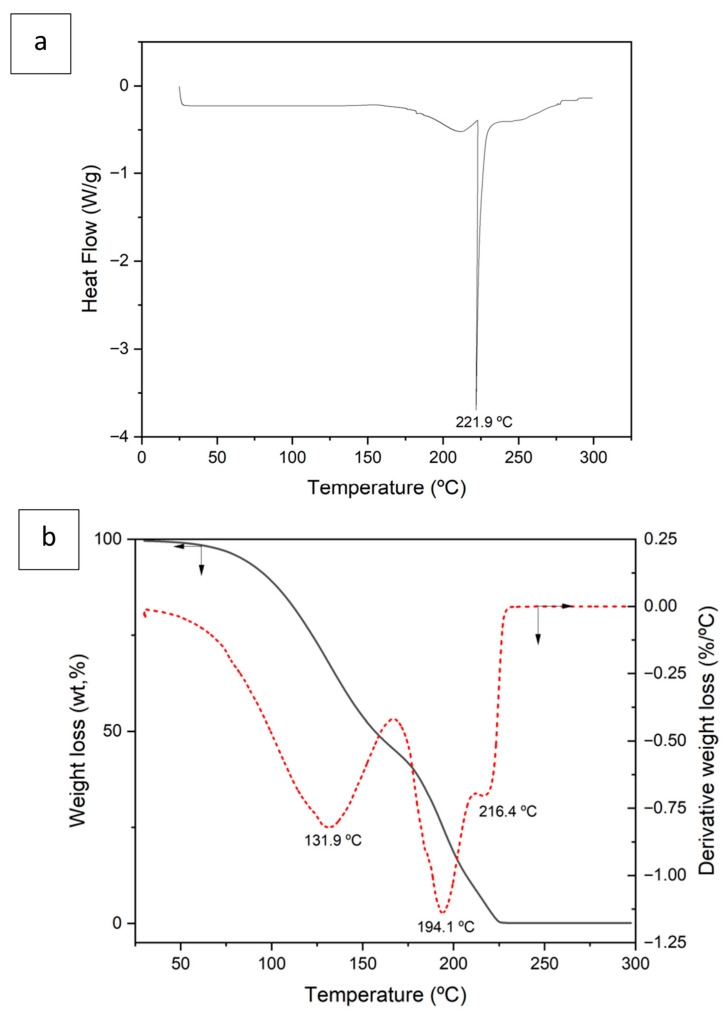
Differential scanning calorimetry (DSC) (**a**) and thermogravimetry (TGA) (**b**) of deep eutectic solvent (DES) (3 Aliquat 336:7 L-Menthol). Red dotted line is the derivate curve.

Figure 2a shows the DSC curve obtained after the formation of DES. An endothermic peak at 221.9 °C with an associated enthalpy of 329.4 J/g is observed, which may correspond to the decomposition of the organic compounds that form the DES (L-Menthol and Aliquat 336).

Figure 2b shows mass loss as a function of temperature (TGA) as well as the derivative of this curve. The TGA curve shows that at 225 °C the total decomposition of the compound occurs, in agreement with the DSC curve data. The total mass loss is 99.6%.

In the derivative curve, three peaks are observed at 131.9 °C, 194.1 °C, and 216.4 °C, respectively. At the temperature range between ambient and 166 °C, a mass loss of 53.81% is observed, which could correspond to the loss of coordination water as well as to the loss of mass corresponding to the initial decomposition of L-menthol [28,29].

Between 166 °C and 211 °C, a 36.4% mass loss is observed, which may correspond to the partial decomposition of the DESs organic constituents, essentially the final decomposition of L-menthol together with the initial decomposition of Aliquat 336 [30,31]. This forms lower-molecular-weight intermediates.

Finally, between 211 °C and 228 °C there is a mass loss of 9.4%, which can be attributed to the decomposition of the intermediate organic products that could have been generated in the previous stage, primarily derived from the earlier decomposition of Aliquat 336.

#### 2.1.3. Nuclear Magnetic Resonance Spectroscopy

**Figure 3:**
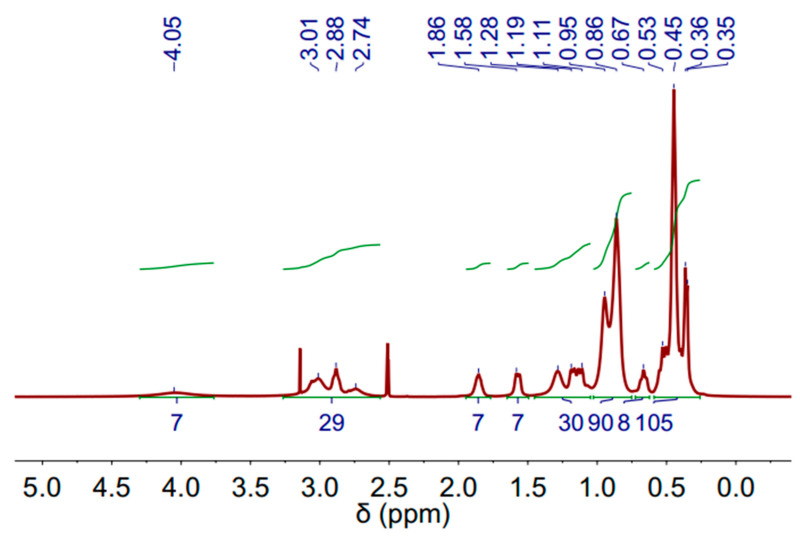
^1^H-NMR spectrum of deep eutectic solvent (DES) (3 Aliquat 336:7 L-Menthol) (500 MHz, DMSO-d6) at 60 °C.

The ^1^H-NMR (Proton Nuclear Magnetic Resonance) spectra of the DES (3 Aliquat:7 L-Menthol) is depicted in Figure 3. The measurements were performed at 60 °C to decrease the sample viscosity and improve the spectra resolution. The molecules and corresponding peaks are assigned in Figure S1 and Table S1. Peaks related to Aliquat-336 and L-Menthol can be observed.

### 2.2. Extraction Experiments with Synthetic Co(II) Solutions

#### 2.2.1. Equilibrium Time

**Figure 4:**
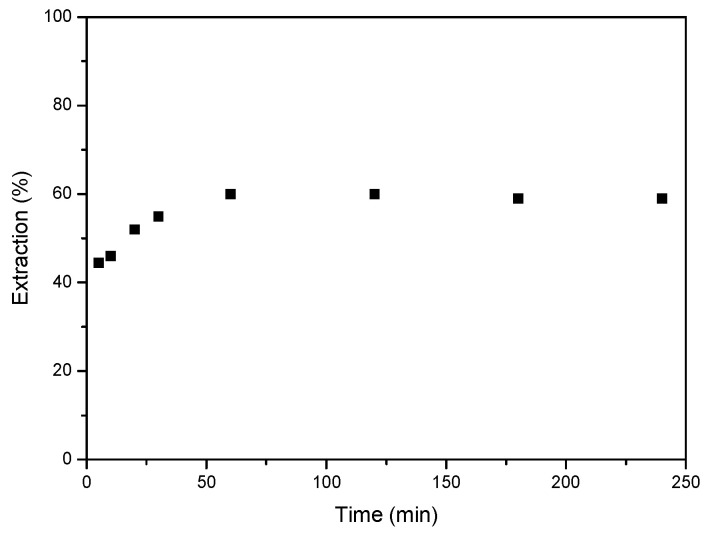
Effect of equilibrium time on the extraction of Co(II). Aqueous phase: [Co]_0_ = 1 g/L Co, 6 M HCl. Organic-to-aqueous ratio 1/1. Temperature 25 °C. Error in all measures: ±0.1.

Initially, the equilibrium time for Co extraction with the deep eutectic solvent was investigated. A series of experiments were conducted in which an aqueous solution containing 1 g/L of Co(II) at a concentration of 6 M HCl was brought into contact with DES. The ratio between the aqueous phase and the organic phase was 1. As shown in Figure 4, equilibrium was reached after 60 min of reaction, with the extraction percentage remaining constant thereafter. The extraction percentages were around 60%, remaining consistent at longer time.

Cobalt distribution coefficients have been studied using Equation (2) at different reaction times (Table 1). The distribution coefficient values range from 1 to 1.44, indicating that cobalt is generally distributed similarly between the aqueous and organic phases (DES), with no significant difference when the reaction time varies.

#### 2.2.2. Influence of Co(II) Concentration on the Extraction of Metal

The effect of the initial Co(II) concentration in the solution was studied in an aqueous phase where the initial (II) concentration ranged from 1 g/L to 6 g/L. The rest of the conditions were kept constant. The results show that the Co(II) extraction, calculated according to Equation (1), is independent of its initial concentration, with extraction percentage values ranging from 55% to 59% for all concentrations studied (Table 2). These findings indicate that an increase in cobalt concentration in the solution does not influence the extraction percentage, suggesting the absence of specimen-contained metal polynuclear species in its composition [32,33]. The coefficient D does not show significant variation with the change in the initial concentration of the metal in solution.

#### 2.2.3. Effect of HCl Concentration on the Aqueous Phase Solution

**Figure 5:**
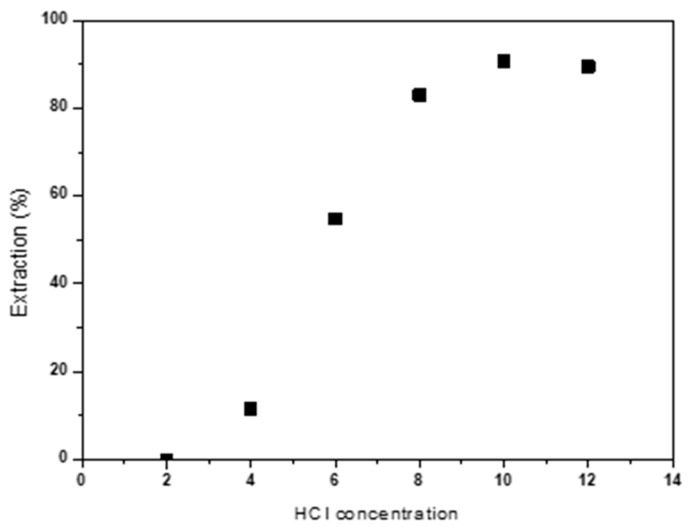
Effect of HCl concentration (M) on Co(II) extraction Aqueous phase: [Co]_0_ = 2 g/L. Temperature: 25 °C. Equilibrium time: 60 min. Error in all measures: ±0.1.

The influence of HCl concentration on Co(II) extraction has also been studied. Extraction tests were conducted varying the acid concentration between 2 and 12M. The obtained results are collected in Figure 5. It is observed that an increase in HCl concentration enhances Co(II) extraction, reaching an extraction percentage of 90% for HCl concentrations of 10 M.

Table 3 shows cobalt distribution coefficients (DCo). The increase in HCl concentration raises the cobalt distribution coefficient between the organic and aqueous phases, reaching the highest values for an HCl concentration of 10 M. Therefore, the extraction of Co(II) is favored in strongly acidic media with high concentrations of Cl- ions, which promote the formation of anionic complex cobalt tetrachloride (CoCl_4_^2−^) [34] according to the reaction (1) [35].

Co(H_2_O)_6_^2+^ + 4Cl^−^ ⇄ CoCl_4_^2−^ + 6H_2_O (1)

The results shown in Table 3 confirm those obtained in Figure 5, where it can be observed that cobalt extraction increases with increasing acidity of the solution, 10 M being the concentration at which the formation of cobalt tetrachloride is most favored.

#### 2.2.4. Two-Stage Co(II) Extraction

To enhance Co extraction yields, a two-stage extraction was carried out. In the second stage, the aqueous phase was retained and mixed with a fresh organic phase. Table 4 presents the results for each individual stage, as well as the total extraction percentage. The study was conducted for two initial Co concentrations, 2 and 6 g/L. Both concentrations exhibited a similar behavior: in the first extraction stage, around 55% of the cobalt in solution was extracted, and in the second stage, the extraction percentages slightly decreased to 50%.

As observed in Table 4, after two stages, the cobalt extraction percentages are approximately 75–78% and appear independent of the initial Co(II) concentration in solution. These percentages are slightly lower than those obtained with higher HCl concentrations (see Section 2.2.3).

#### 2.2.5. Influence of Phase Relationship on the Extraction of Metal

Various phase ratios have been studied and their influence on the extraction process investigated. The results obtained are shown in Table 5. It is observed that an increase in the aqueous–organic ratio from 0.33 to 2 reduces the recovery percentages and the distribution coefficient values, decreasing from 73% to 52%.

#### 2.2.6. Nuclear Magnetic Resonance Spectroscopy of DES After Extraction Process

**Figure 6:**
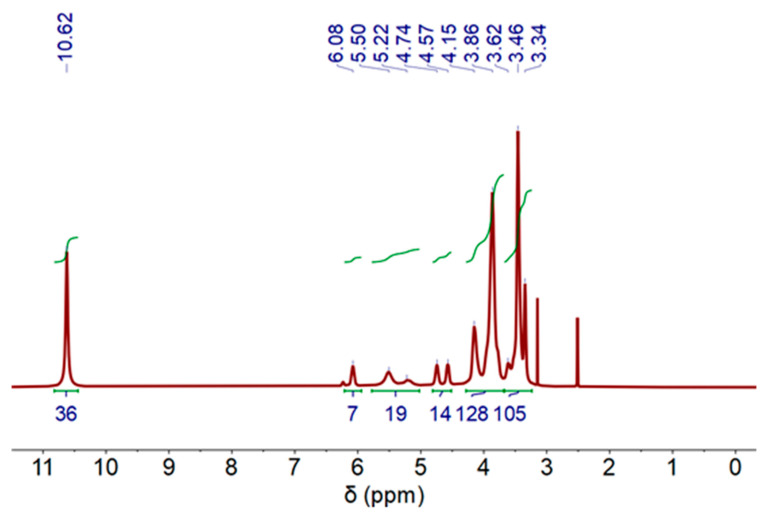
^1^H-NMR spectra of deep eutectic solvent (DES) after Co extraction process, (500 MHz, DMSO-d6) at 60 °C.

^1^H-NMR was measured in the organic phases (DES-phase) after cobalt extraction process, and results are shown in Figure 6. As can be appreciated, a similar NMR profile was registered. However, the presence of Co(II) causes the signals to shift toward lower fields compared to DES displacements without Co(II) (see Figure 3). It should be noted that only some peaks can be assigned in the present sample (see Figure S1 and Table S1).

### 2.3. Stripping Experiments

The stripping experiments were conducted by loading the organic phase with a solution of 2 g/L of Co(II) and stripping it with various acids (HCl and H_2_SO_4_) at different concentrations. Concentrations of 0.01 and 0.1 M of HCl, as well as 0.01, 0.5, 1, 3, and 5 M of H_2_SO_4_, were used. The experiments were carried out for 60 min at 25 °C. The organic phase-to-re-extraction phase ratio was set at to 1.

Table 6 shows the obtained results using HCl. For both concentrations, it is possible to recover 100% of the cobalt from the organic phase, indicating that the HCl concentration does not influence the stripping of Co(II).

In the case of H_2_SO_4_, the obtained results show that increasing acid concentration decreases the recovery percentages. It ranges from 100% at 0.01 M to 58% at 5 M (Table 7).

#### Nuclear Magnetic Resonance Spectroscopy of DES After Stripping Process

**Figure 7:**
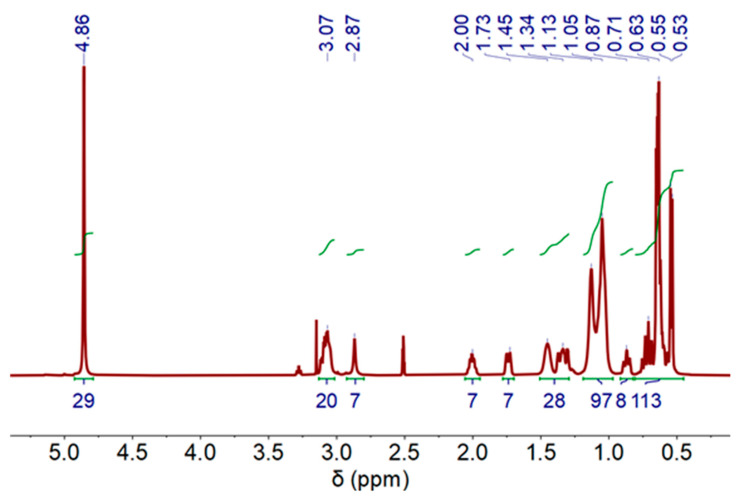
^1^H-NMR spectra of deep eutectic solvent (DES) after Co stripping process, (500 MHz, DMSO-d6) at 60 °C.

Regarding the stripping process, Figure 7 shows the ^1^H-NMR measurement spectrum for the DES-phase. A minimal chemical downfield shift compared to the initial DES can be observed (see Figure S1 and Table S1), indicating that some cobalt content could remain in the DES (not fully recovered: Co stripping (%) = 84%).

In order to obtain Co(II) salts, the stripping aqueous phases obtained with 0.01 M and 0.5 M H_2_SO_4_ (where the Co(II) stripping percentages are 100% and 84%, respectively) were evaporated and dried at 80 °C for 24 h. The obtained solids were characterized using X-ray diffraction (XRD).

**Figure 8:**
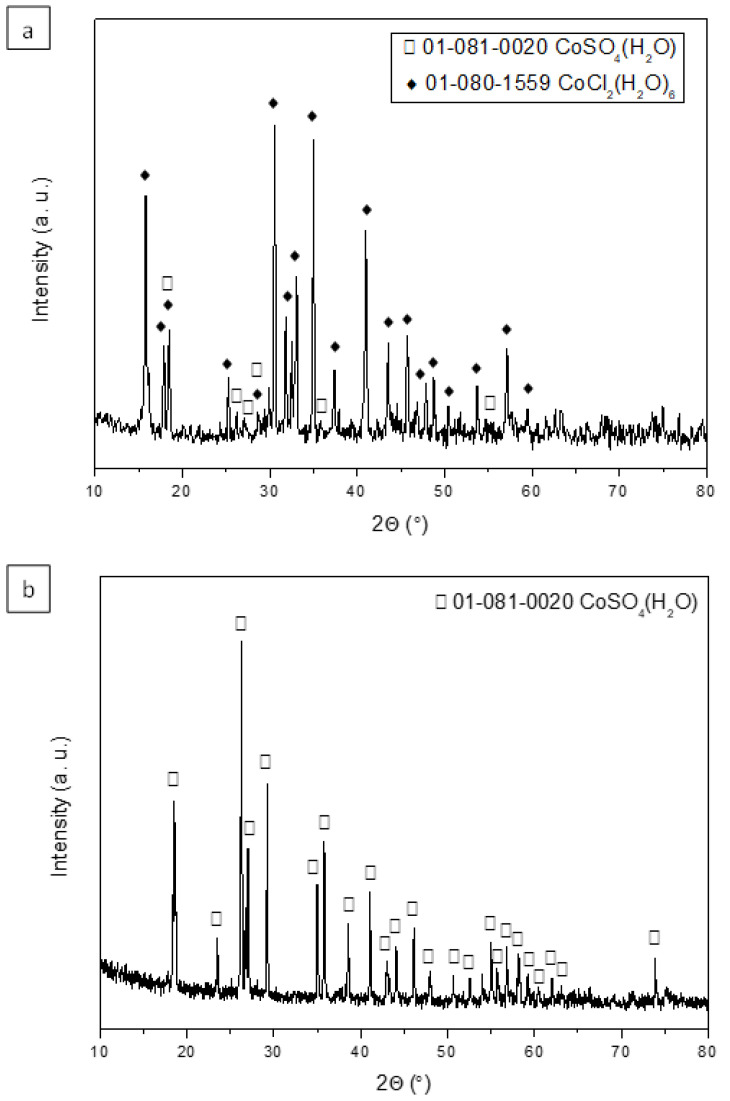
X-ray diffraction patterns of the salts obtained using a H_2_SO_4_ concentration in the stripping stage of (**a**) 0.01 M and (**b**) 0.5 M.

Figure 8a shows the diffractogram of the salt obtained using a stripping phase with a concentration of 0.01 M H_2_SO_4_. The main diffraction peaks indicate that the predominant crystalline phase is cobalt chloride hexahydrate, with minor presence of cobalt sulfate monohydrate. However, when using a stripping aqueous phase with a concentration of 0.5 M H_2_SO_4_, the obtained salt corresponds to cobalt sulfate monohydrate (Figure 8b).

### 2.4. Reuse of the DES

An important aspect in liquid–liquid extraction is studying the reuse of the organic phase. For this purpose, two complete cycles of extraction and stripping were carried out maintaining the same organic phase. Table 8 displays the extraction stripping percentages as well as the cobalt distribution coefficients using initial Co(II) concentrations of 2 and 6 g/L. It is observed that after the first cycle of extraction and stripping, the DES loses efficiency, decreasing the extraction percentage by approximately half, regardless of the cobalt concentration used in the test.

This loss of DES efficiency after the first cycle may be due to the possible saturation of the DES, which reduces its capacity to extract Co(II) from the aqueous phase. In addition, absorption of small amounts of aqueous phase into the DES could contaminate the organic phase and reduce its efficiency [20]. 

### 2.5. Optimal Conditions for Cobalt Recovery

The obtained results indicate that the optimal conditions for Co(II) extraction are achieved in a single extraction and stripping stage at an initial concentration of 6 g/L Co(II), 10 M HCl, an aqueous-to-organic phase ratio of 1/1, a temperature of 25 °C, and an equilibrium time of 60 min. Stripping using 0.5 M H_2_SO_4_ at temperature of 25 °C with an equilibrium time of 60 min provides the best results. Table 9 displays the extraction and stripping percentages as well as the distribution coefficients under the optimal conditions. The Co(II) recovery reaches 91% in the extraction stage and 84% in the stripping stage.

### 2.6. Extraction of Co(II) in Black Masses of NMC 622 Batteries

To investigate the selectivity of the DES toward other metals present in leaching solutions of black masses from NMC 622-type lithium-ion batteries, extraction experiments were conducted under the optimal conditions described in Section 2.5.

Table 10 displays the concentrations of different metals in the solutions obtained after leaching the black masses.

Table 11 and Table 12 show the extraction and stripping percentages for each analyzed element in the studied black mass samples.

Table 11 shows that the extraction is selective toward cobalt and copper, with extraction percentages > 80% for Co(II) and >79.5% for Cu(II) in all the studied samples. In contrast, Ni and Li are not extracted in any of the samples.

In the stripping process (Table 12) using 0.5 M H_2_SO_4_, a higher selectivity for Co(II) over Cu(II) is observed, with stripping percentages > 76% for Co(II) and <57% for Cu(II).

**Figure 9:**
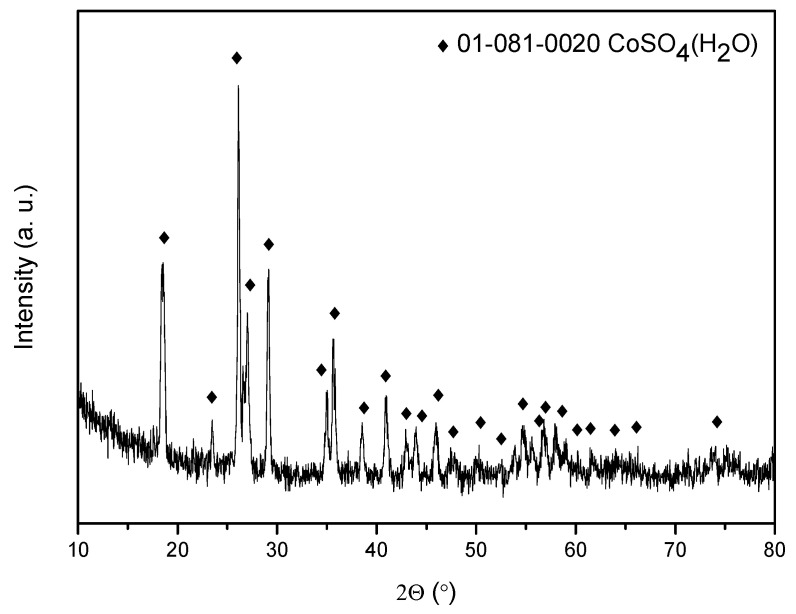
X-ray diffraction pattern of the obtained solid (Aqueous Phase: 0.5 M H_2_SO_4_) from the treatment of BM6.

The evaporation of the stripping aqueous phases leads to the formation of cobalt sulfate monohydrate as the major salt, as revealed by its X-ray diffraction diagram (Figure 9). The figure shows the XRD diagram for sample BM6 as an example. For the other samples, the diagram is identical to that for sample BM6.

It is observed that some residual manganese is extracted during the extraction process in some of the samples. The extraction behavior of manganese could be influenced by its concentration in the leached solution relative to cobalt. As can be observed in Table 11, in general, when the concentration of Mn exceeds that of Co in the leached solution, competitive extraction of Mn becomes more pronounced. However, when Mn and Co are present in similar amounts, Mn extraction remains minimal. These trends could suggest that Mn competes with Co when its concentration approaches or exceeds that of Co. The optimization of this Mn extraction is a new ongoing research project that will be the subject of further work in the near future.

Based on the results, the process conditions should be optimized to improve selective recovery of Co(II) over Cu(II).

### 2.7. Optimization of the Extraction Conditions of the Co/Cu Separation Process

#### 2.7.1. Separation of Cu(II) from Synthetic Solutions and Black Mass Leaching Solutions Containing Co(II), Mn(II), Ni(II), and Li(I)

**Figure 10:**
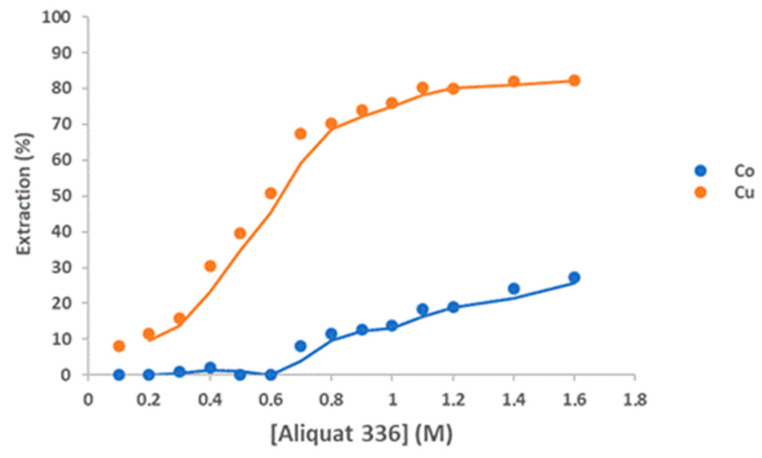
Effect of the Aliquat 336 concentration on the extraction of Cu(II) from a 3 M HCl solution; Aqueous phase: [Co]_0_ = 5 g/L, [Cu]_0_ = 6 g/L, Aqueous-to-organic ratio 1/1; Temperature: 25 °C. Equilibrium time: 60 min. Error in all measures: ±0.1.

In an HCl solution, metal ions form complexes with chloride ions. To utilize thedifference in complex formation tendency among the metals ions, Aliquat 336 with 10% *v*/*v* decanol as a modifier was employed for the selective extraction of Cu(II) from the solution. Figure 10 shows that the extraction percentage of Cu(II) increased from 8.2 to 82.3% as the Aliquat 336 concentration increased from 0.1 to 1.6 M. When the Aliquat 336 concentration was 0.6 M, Co(II) began to be co-extracted with Cu(II) (50.9%) and 27.3% of Co(II) was extracted by 1.6 M Aliquat 336. On the other hand, when Aliquat 336 concentration was 0.7 M, 8% of Co(II) was extracted with 67.5% of Cu(II). The extraction of Cu(II) from 3 M HCl solution by Aliquat 336 can be represented as follows [36]:

CuCl_4_^2−^(a) + 2[R_3_R’NCl](o) = [(R_3_R’N)2CuCl_4_](o) + 2Cl^−^(a)

where R = C8 aliphatic and R’ = methyl.

##### Equilibrium Time

**Figure 11:**
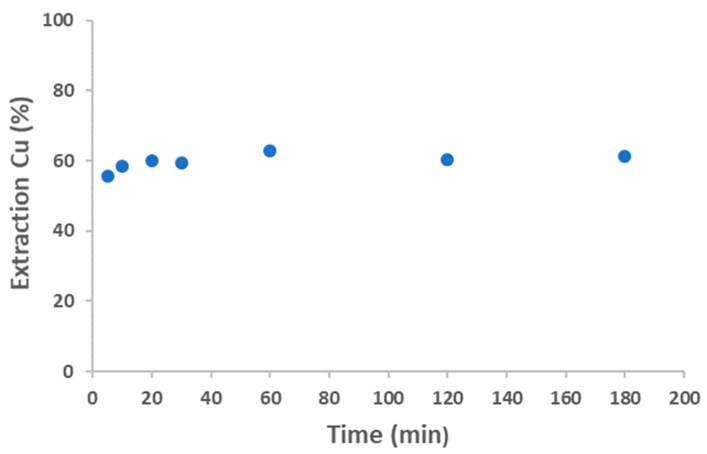
Effect of equilibrium time on the extraction of Cu(II). Aqueous phase: [Cu]_0_ = 6 g/L Co, [Cu]_0_ = 6 g/L Cu, 3 M HCl. Organic-to- aqueous ratio 1/1. Temperature 25 °C. Error in all measures: ±0.1.

Initially, the equilibrium time for Cu extraction with Aliquat 336 was investigated. A series of experiments were conducted in which an aqueous solution containing 5 g/L of Co(II) and 6 g/L of Cu(II) in 3 M HCl was brought into contact with Aliquat 336. The ratio between the aqueous phase and the organic phase was 1. As shown in Figure 11, equilibrium was reached at 60 min of reaction, with the extraction percentage remaining constant thereafter.

##### Extraction Stages

Table 13 shows that four stages of extraction are required for complete extraction of Cu(II) from the aqueous solution containing 5 g/L of Co(II) and 6 g/L of Cu(II), obtaining 97.5% Cu(II) extraction over the for stages using 0.7 M Aliquat 336 and 95.1 with 0.7 M Aliquat 336. Using 0.6 M of Aliquat, the Co(II) extraction is zero.

##### Copper Stripping

The stripping experiments were conducted by loading the organic phase with an aqueous solution containing 5 g/L of Co(II) and 6 g/L of Cu(II) under the same conditions as the previous experiments (organic phase: Aliquat 336 0.7 M in kerosene with 10% decanol) with H_2_SO_4_ 1 M. The loaded organic phase was stripped with 1 M H_2_SO_4_. The reactions were carried out for 60 min at a temperature of 25 °C. The organic phase to stripping phase ratio was set at 1. Experimental results indicated that Cu(II) was completely stripped by 1 M H_2_SO_4_ solution.

The copper extraction process studied has been applied to the recycling of Li-ion batteries NMC 622, using aqueous solutions from the leachate of two black masses of these batteries in 3 M HCl. Table 14 displays the concentrations of the studied metals in the solutions obtained after leaching the two black masses. Concentration of the studied metals in the solutions.

Table 15 and Table 16 show the extraction percentages for two leached black masses using Aliquat 336 0.6 M or 0.7 M in kerosene.

Table 15 shows that four stages of extraction are required for complete extraction of Cu(II) from the aqueous solution of black mass BM5, obtaining 99.5% Cu(II) extraction using 0.6 M Aliquat 336. Table 16 shows that four stages of extraction are sufficient to extract Cu(II) using 0.7 M Aliquat 336 as an extraction agent, obtaining 99.8% Cu(II) extraction.

In the case of sample TUC2, four stages of extraction are necessary to extract copper, reaching an extraction rate of 100% of the copper using Aliquat 336 at 0.7 M concentration as an extraction agent. The same percentage is obtained when the extraction agent is used at a concentration of 0.6 M (Table 16).

The most favorable conditions chosen for the extraction of Cu(II) with the lowest extraction of Co(II) are as follows: organic phase: Aliquat 336 0.6 M in kerosene with 10% decanol. Aqueous-to-organic ratio 1/1. Temperature 25 °C, with two extraction stages.

##### Cu Extraction/Stripping Process in 3 M HCl Medium

The Cu(II) in solution was extracted in a 3 M HCl medium with an organic phase Aliquat 336 0.6 M in kerosene with 10% decanol. The aqueous-to-organic ratio was 1/1, the temperature was 25 °C and equilibrium time was 60 min.

Table 17 shows that two stages of extraction are required for complete extraction of Cu(II) from the aqueous solution of black mass BM5 (in medium 3 M HCL) obtaining 92% Cu(II) extraction using 0.6 M Aliquat 336 as the extractant.

In the case of the TUC2 sample with two extraction stages, 94% of the copper existing in the aqueous leaching phase is removed. It can be seen in the table that a minority percentage of Co(II) is also extracted.

Two extraction stages were chosen to extract the copper in solution, instead of the four phases carried out previously (Table 17), to avoid the extraction of Co while still maintaining a good percentage of copper removal.

The stripping experiments on the Cu(II) contained in the organic phases were conducted using 1 M H_2_SO_4_. The reactions were carried out for 60 min at 25 °C. The organic phase to tripping phase ratio was set at one.

Table 18 shows that Cu(II) can be stripped by 1 M H_2_SO_4_ solution in the two studied black mass samples.

##### Recovery of Co(II) Using DES 3 Aliquat 336:7 L-Menthol

Once the Cu(II) in solution had been removed, the extraction of the Co(II) contained in solution was carried out. This metal was recovered in 10 M HCl medium (to enhance the formation of anionic complexes of Co(II)) with the DES 3 Aliquat 336:7 L-Menthol. To carry out the test in 10 M HCl medium, the samples (in 3 M HCl medium) were concentrated in HClcc in a ratio of 1/5: sample/HClcc. The aqueous-to-organic ratio was 1/1, the temperature was 25 °C and equilibrium time was 60 min.

Table 19 shows that the extraction percentage was 93% for Co(II) in the two of the studied samples. The concentration of copper in the aqueous extraction phases was practically negligible (0.003 and 0.5 × 10^−3^ g/L) due to its elimination in the previous phase of the process.

Table 20 shows that experimental results indicated that Co(II) can be stripped by 0.5 M H_2_SO_4_ solution in the two studied black mass samples, with stripping percentages > 82% and >71%, respectively.

From an economic and environmental viewpoint, menthol-based DESs constitute a cost-effective and sustainable alternative to conventional organophosphorus extractants. The raw materials (menthol and organic acids) are inexpensive (<10 €/kg) and renewable, while commercial extractants such as Cyanex 272 or D2EHPA cost around 70–100 €/kg and require additional organic diluents. Furthermore, the DES phase can be regenerated and reused in at least five extraction–stripping cycles with negligible efficiency loss, considerably reducing operational costs and solvent waste. The non-volatile and biodegradable nature of these DESs further enhances their environmental performance, supporting their practical applicability in large-scale cobalt recovery from NMC battery leachates.

## 3. Materials and Methods

### 3.1. Chemical Reagents

The aqueous solutions were prepared with the following reagents: cobalt(II) sulfate heptahydrate (CoSO_4_·7H_2_O) and copper(II) sulfate pentahydrate (CuSO_4_·5H_2_O), supplied by Sigma Aldrich, and hydrochloric acid 37% (HCl) supplied by Panreac AppliChem. DES was prepared using Aliquat 336 ([CH_3_(CH_2_)_7_]_3_NCH_3_Cl) and L-menthol 99% (C_10_H_20_O) supplied by Thermo Fisher Scientific (Waltham, MA, USA).

### 3.2. Synthesis and Characterization of DES

The synthesis of the DES (3 Aliquat: 7 L-Menthol) was carried out by mixing the two respective components (Aliquat 336:L-menthol, molar ratio 3:7) under continuous stirring (at 300 rpm) for 30 min at 60 °C. The components were placed in an Erlenmeyer flask and immersed in a silicone oil bath with immersion thermostat (model DIGITERM TFT-200, J.P. SELECTA, Scharlab S.L, Barcelona, Spain). The reaction was maintained until a clear and homogenous liquid was obtained [37]. The formation of the DES was studied by Fourier-transform infrared spectroscopy (FTIR) [14]. 

The infrared spectra of the DES synthesized as well as the initial reagents, were obtained using a Nicolet iS50 FT-IR Spectrometer of Thermo Scientific^TM^, operated in Attenuated Total Reflection (ATR) mode, in the range from 4000 to 400 cm^−1^, with spectral resolution of 4 cm^−1^ and accumulation of 64 scans.

The thermal analysis study of the DES was synthesized (differential scanning calorimetry (DSC) and thermogravimetry (TGA)) was carried out in a differential scanning calorimeter model DSC 25 and a thermogravimetric analyzer model of TGA 550 from TA instrument (Lukens Drive, New Castle, DE, USA).

^1^H-NMR spectra of the DES was synthesized and the DES-Co were conducted using a Bruker Avance DRX500 (NMR Technologies, San Francisco, CA, USA) spectrometer operating at 500 MHz. The NMR parameters employed included a 30° pulse, an acquisition time of 3.1719 s, a relaxation delay of 1 s, and a total of 16–32 scans. All samples were placed in capillary tubes inside 5 mm NMR glass tubes and analyzed using dimethyl sulfoxide (DMSO-d6) as an external reference. The spectra were acquired after setting the temperature to 25 or 60 °C using a Bruker Variable Temperature BVT (NMR Technologies, San Francisco, CA, USA). 

Measurements of the density of the DES was made by difference in weight in an Ohaus Explorer Analytical 110G Balance (Sigma Aldrich, Madrid, Spain), for which six measurements were carried out, and the standard deviation of the samples was calculated. The density of the DES was measured at 25 °C. The density obtained was 0.886 ± 0.003 g/mL.

The viscosity of the DES was determined using a viscometer PCE-RVI2 (PCE Instrument, Tobarra, Spain). The analysis was performed at 20 °C. Temperature control was carried out in a water bath. Four measurements were carried out in which the standard deviation and error of the samples were calculated. The medium viscosity obtained was 608.90 ± 0.03 cp.

### 3.3. Preparation of Solutions from Black Masses

Six black masses, provided by a Spanish company, have been employed to study the selective separation of Co(II). BM6, BM8, and BM9 are black masses originating from the crushing, magnetic separation, and eddy current separation of NMC 662-type battery cells. Samples BM5, BM1, and TUC2 come from carbo-pyrometallurgical processes for pre-recovery of Li [38]. The samples were leached using a 10 M HCl solution (black mass/hydrochloric acid ratio S/L = 100 g/L; temperature = 70 °C, re-action time = 2 h). The resulting suspensions were filtered, and the solutions were treated with the DES (3 Aliquat 326:7 L-Menthol) utilized for this study. The preliminary extraction of Cu(II) contained in the black masses (samples BM5 and TUC2), leached using a 3 M HCl solution, was carried out with Aliquat 336 in kerosene.

### 3.4. Extraction Experiments

The extractions of the metals (Co/Cu) were carried out with aqueous solutions of fixed concentration for each test in a shaking funnel with the corresponding amount of DES (3 Aliquat 326:7 L-Menthol) (no solvent was used in the test) or Aliquat 336 in kerosene (with 10% decanoic acid). Solvent extractions were carried out by stirring the mixture for different lengths of time (equilibrium time = 60 min). The temperature was maintained at a constant 25 °C during all experiments. The metal concentration in the aqueous solution was analyzed by aqueous adsorption spectrophotometry in an atomic absorption spectrophotometer (AAS), model contraAA 800 of Analytik Jena, Jena, Germany.

The metal concentration in the organic phase was estimated by mass balance. In the stripping stage, the aqueous phase was analyzed by AAS, and the concentration of metal remaining in the organic phase was also determined by mass balance.

The metal (*M*) extraction percentage is obtained by Equation (2):

(2) $$\%E=\;\frac{{\left[M\right]}_{Org}\times \;V_{Org}}{{\left[M\right]}_{Aq0}\times \;V_{Aq0}}\;\times \;100$$

where [*M*]*_org_* corresponds to the metal concentration in the organic or DES phase after the equilibration time and [*M*]*_Aq_*_0_ corresponds to the cobalt concentration in the initial aqueous phase. *V_Org_* and *V_Aq_*_0_ are, respectively, the volume in the organic phase or DES phase after the equilibration time and in the initial aqueous phase.

The cobalt distribution coefficient is defined as Equation (3):

(3) $$D_{Co}=\;\frac{{\left[M\right]}_{Org}}{{\left[M\right]}_{Aq}}$$

where [*M*]*_Org_* corresponds to the M concentration in the organic phase or DES and [*M*]*_Aq_* indicates the cobalt content in the aqueous phase, both after the equilibrium time.

The stripping percentages were calculated according to the following equation:

(4) $$\%E=\;\frac{{\left[M\right]}_{Aqrex}\times V_{Aqrex}}{{\left[M\right]}_{Org}\;\times \;V_{Org}}\;\times \;100$$

${\left[M\right]}_{Aqrex}$, ${\left[M\right]}_{Org}$ corresponds to the concentration of metal in the stripping aqueous phase and in the organic phase, respectively. *V_Aqrex_* and *V_Org_* are, respectively, the volume in the stripping aqueous phase and in the organic phase.

### 3.5. Schemes of the Studied Extraction/Re-Extraction Processes

**Figure 12:**
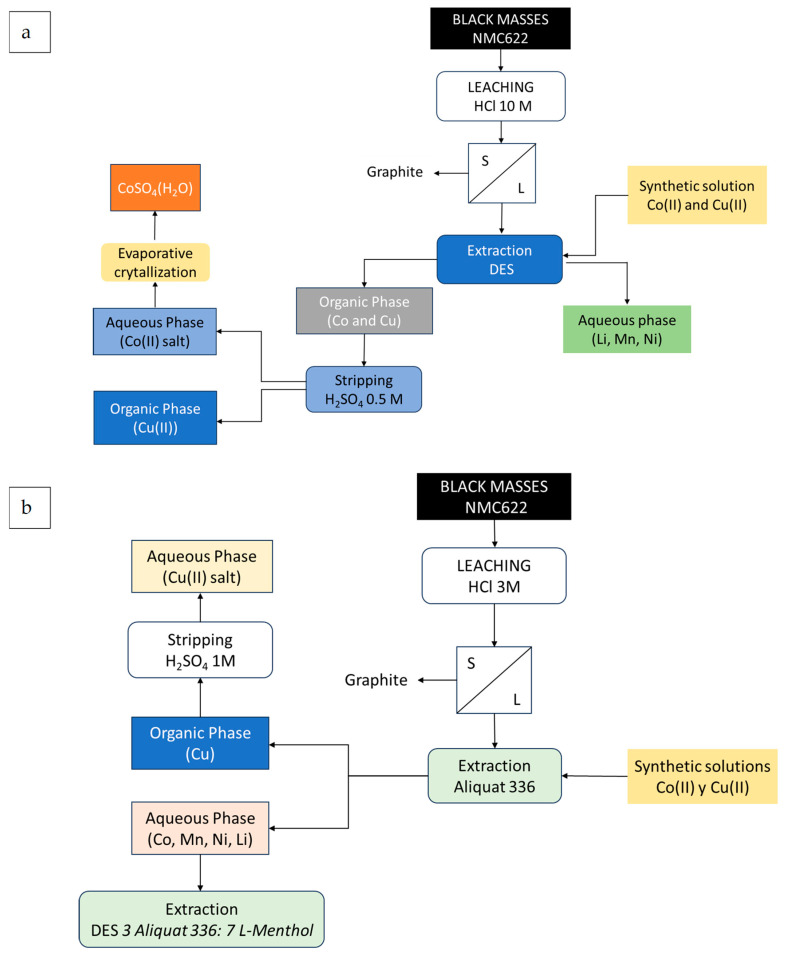
Extraction and stripping of Co(II) (**a**). Separation of Cu(II) (**b**) from synthetic solutions and black mass leaching solutions.

Figure 12a shows schemes of the studied processes, starting from both synthetic solutions and black masses.

### 3.6. Synthesis of Cobalt(II) Salts

The stripping solutions were evaporated in a BUCHI R-100 Rotavapor (BUCHI, Barcelona, Spain) equipped with a V-100 Vacuum Pump and a B-100 Heating Bath. The solids obtained were dried in an oven (80 °C, 24 h) and their mineralogical characterization was performed by X-ray diffraction using an X’Pert Pro MPD diffractometer (PANalytical) (PANalytical Almelo, the Netherlands) equipped with a Cu anode (Cu K radiation) with a step size of 0.03° (2Ɵ) in the range 10–80.

## 4. Conclusions

The DES used in this work (3 Aliquat 336:7 L-Menthol) achieved a cobalt extraction of 91% (experimental conditions: 6 g/L Co, 10 M HCl, teq = 1 h, Aqueous phase/Organic phase = 1, T: 25 °C). The extraction of Co is favored in strongly acidic media with high concentrations of Cl- ions, which promote the formation of anionic complex cobalt tetrachloride (CoCl_4_^2−^). The extraction of cobalt contained in the black masses studied was 88% (sample BM8) after a leaching process in a hydrochloric medium and liquid–liquid extraction under the most favorable conditions tested, managing to re-extract 85% (sample BM1) of the cobalt contained in the organic phase or DES. Cobalt is recovered as CoSO_4_(H_2_O) using a 0.5 M H_2_SO_4_ stripping solution. DES 3 Aliquat 336:7 L-Menthol is selective in extracting Co contained in the black masses of NMC batteries compared to other elements present in these samples (Li, Ni and Mn). Based on the results obtained, the process has been optimized to achieve greater selectivity in the recovery of Co(II) over Cu(II), achieving the prior separation of Cu(II) in 3 M HCl medium (94% extraction in sample TUC2) using Aliquat 336 in kerosene. Once copper has been removed from the sample, cobalt is extracted under the most favorable conditions studied, obtaining a Co(II) extraction of 93% with stripping > 82% (sample BM5). Liquid–liquid extraction using the studied DES (3 Aliquat 336:7 L-Menthol) as an extraction agent is a very promising method for Co recovery from lithium batteries NMC 622, Co being a metal that is currently in high demand for the energy transition. It offers advantages over liquid–liquid extraction with organic solvents and extraction agents due to its lower environmental impact and high percentage of metal recovery. Therefore, L-Menthol-based DES will represent a breakthrough in cobalt extraction compared to other less environmentally friendly processes, with the consequent advance that this represents compared to other processes used previously.

It is observed that some residual manganese is extracted during the extraction process in some of the samples. The extraction behavior of manganese could be influenced by its concentration in the leached solution relative to cobalt. Optimizing this process is an ongoing research project that will be the subject of new work in the near future.

## Figures and Tables

**Table 1:** Distribution coefficients as a function of reaction time.

Reaction Time (Min)	DCo
5	0.955 ± 0.020
10	1.064 ± 0.010
20	1.108 ± 0.030
30	1.200 ± 0.010
60	1.464 ± 0.020
120	1.443 ± 0.0030
180	1.344 ± 0.0020
240	1.333 ± 0.0010

**Table 2:** Influence of initial Co(II) concentration on the extraction of metal.

[Co]_0_ (g/L)	E Co (%)	DCo
1	57 ± 0.1	1.344 ± 0.002
2	55 ± 0.1	1.207 ± 0.010
4	59 ± 0.1	1.410 ± 0.003
6	55 ± 0.1	1.235 ± 0.010

Aqueous phase: [Co]_0_ 1 and 6 g/L de Co, 6 M HCl, Aqueous-to-organic ratio 1/1. Temperature: 25 °C, Equilibrium time: 60 min.

**Table 3:** Influence of initial HCl concentration on the extraction process.

[HCl]_0_ (M)	[Cl^−^] (M)	DCo
2	4.472 × 10^3^	0.000 ± 0.020
4	6.324 × 10^3^	0.130 ± 0.020
6	6.324 × 10^3^	1.207 ± 0.010
8	8.944 × 10^3^	4.896 ± 0.030
10	1.095 × 10^4^	9.794 ± 0.040
12	1.095 × 10^4^	8.489 ± 0.020

**Table 4:** Results obtained in the two consecutive stages of Co(II) extraction.

(a) [Co]_0_ = 2 g/L		
Stage	E Co (%)	DCo
1	54 ± 0.1	1.207 ± 0.010
2	52 ± 0.1	1.073 ± 0.008
Total	75 ± 0.1	4.833 ± 0.030
**(b) [Co]_0_ = 6 g/L**		
**Stage**	**E Co (%)**	**D** **Co**
1	55 ± 0.1	1.235 ± 0.010
2	53 ± 0.1	1.111 ± 0.009
Total	78 ± 0.1	4.962 ± 0.020

Aqueous phase: 6 M HCl, Temperature: 25 °C, Aqueous-to-organic ratio 1/1. Equilibrium time: 60 min.

**Table 5:** Influence of the aqueous–organic phase ratio on Co(II) extraction.

Aqueous-to-Organic Ratio	E Co (%)	DCo
10/30	73 ± 0.1	2.770 ± 0.002
10/20	59 ± 0.1	1.454 ± 0.001
20/20	55 ± 0.1	1.207 ± 0.002
20/10	52 ± 0.1	1.141 ± 0.004

Aqueous phase: [Co]_0_ 2 g/L, 6 M HCl, Temperature: 25 °C, Equilibrium time: 60 min.

**Table 6:** Influence of the concentration of the stripping solution (HCl) on the percentage of recovered Co(II).

HCl [M]	Co Stripping (%)
0.01	100 ± 0.1
0.1	100 ± 0.1

Organic phase: [Co] 2 g/L de Co; Aqueous-to-organic ratio: 1/1; Temperature: 25 °C, Equilibrium time: 60 min.

**Table 7:** Influence of the concentration of the stripping aqueous solution (H_2_SO_4_) on the percentage of recovered Co(II).

H_2_SO_4_ [M]	Co Stripping (%)
0.01	100 ± 0.1
0.5	84 ± 0.1
1	84 ± 0.1
3	76 ± 0.1
5	58 ± 0.1

Organic phase: [Co]: 2 g/L of Co(II), Aqueous-to-organic ratio: 1/1 Temperature: 25 °C, Equilibrium time: 60 min.

**Table 8:** Extraction and stripping percentages of Co(II) and distribution coefficients after two consecutive stages, for different Co(II) concentrations.

(a) [Co]_0_ = 2 g/L		
	E Co (%)	DCo
**1st cycle**		
Extraction	65 ± 0.1	1.839 ± 0.001
Stripping	91 ± 0.1	10.560 ± 0.020
**2nd cycle**		
Extraction	35 ± 0.1	0.546 ± 0.002
Stripping	100 ± 0.1	-
**(** **b) [Co]_0_ = 6 g/L**		
	**E Co (%)**	**D** **Co**
**1st cycle**		
Extraction	60 ± 0.1	1.520 ± 0.002
Stripping	91 ± 0.1	10.071 ± 0.030
**2nd cycle**		
Extraction	37 ± 0.1	0.597 ± 0.004
Stripping	100 ± 0.1	-

Aqueous phase: 6 M HCl, Aqueous-to-organic ratio: 1/1; Temperature: 25 °C, Equilibrium time: 60 min, and stripping phase 0.01 M H_2_SO_4_, Equilibrium time 60 min.

**Table 9:** Percentages and distribution coefficients under the best conditions of extraction and stripping stages of Co(II).

	E Co (%)	DCo
Extraction	91 ± 0.1	9.998 ± 0.030
Stripping	84 ± 0.1	5.438 ± 0.020

**Table 10:** Concentration of the studied metals in the solutions after leaching in HCl medium (10 M) of the studied black masses, ratio S/L = 100 g/L, reaction time = 2 h, temperature = 70 °C. Error in all measures: ±0.1.

Metal	BM6 (g/L)	BM8 (g/L)	BM9 (g/L)	BM5 (g/L)	BM1 (g/L)	TUC2 (g/L)
Co	10.3	7.3	12.1	4.4	12.9	9.0
Ni	33.8	12.8	50.4	24.4	27.7	27.3
Mn	10.3	13.8	13.9	4.5	10.2	8.1
Cu	1.2	7.3	8.7	3.8	1.1	0.9
Li	6.6	2.8	1.2	0.7	1.3 × 10^−3^	2.3
IR *	31.3	32.6	18.0	53.9	41.3	53.9

* IR = Insoluble residue.

**Table 11:** Extraction percentages for leached black masses.

Metal	E (BM6) (%)	E (BM8) (%)	E (BM9) (%)	E (BM5) (%)	E (BM1) (%)	E (TUC2) (%)
Co	80.2 ± 0.1	87.8 ± 0.1	81.2 ± 0.1	84.7 ± 0.1	83.1 ± 0.1	83.9 ± 0.1
Ni	0	0	0	0	0	0
Mn	2.9 ± 0.1	14.9 ± 0.1	0	8.8 ± 0.1	0	0
Cu	87.1 ± 0.1	92.5 ± 0.1	84.4 ± 0.1	83.3 ± 0.1	85.3 ± 0.1	79.5 ± 0.1
Li	0	0	0	0	0	0

**Table 12:** Stripping percentages for leached black masses (with 0.5 M H_2_SO_4_).

Metal	RE (BM6) (%)	RE (BM8) (%)	RE (BM9) (%)	RE [BM5) (%)	RE (BM1) (%)	RE (TUC2) (%)
Co	82.7 ± 0.1	75.8 ± 0.1	79.7 ± 0.1	100 ± 0.1	84.8 ± 0.1	86.9 ± 0.1
Ni	0	0	0	0	0	0
Mn	100 ± 0.1	100 ± 0.1	0	100 ± 0.1	0	0
Cu	19.8 ± 0.1	18.7 ± 0.1	39.6 ± 0.1	57.6 ± 0.1	31.6 ± 0.1	51.5 ± 0.1
Li	0	0	0	0	0	0

**Table 13:** Stages of extraction for removal of Cu(II). Aqueous phase: [Co]_0_ = 5 g/L, [Cu]_0_ = 6 g/L, 3 M HCl. Organic phase: Aliquat 336 0.6/0.7 M in kerosene with 10% decanol. Aqueous-to-organic ratio 1/1. Temperature 25 °C.

Aliquat 336 (M)	Stage	(Co)_AP_ * (g/L)	(Cu)_AP_ * (g/L)	E Co (%)	E Cu (%)
0.6	1	-	3.0 ± 0.1	0	54.1 ± 0.1
0.6	2	-	1.4 ± 0.1	0	78.4 ± 0.1
0.6	3	-	0.7 ± 0.1	0	88.7 ± 0.1
0.6	4	-	0.3 ± 0.1	0	95.1 ± 0.1
0.7	1	4.5 ± 0.1	1.9 ± 0.1	12.9 ± 0.1	67.5 ± 0.1
0.7	2	4.5 ± 0.1	0.7 ± 0.1	11.5 ± 0.1	88.3 ± 0.1
0.7	3	4.0 ± 0.1	0.3 ± 0.1	20.9 ± 0.1	95.1 ± 0.1
0.7	4	3.7 ± 0.1	0.2	27.1 ± 0.1	97.5 ± 0.1

* AP = aqueous phase.

**Table 14:** Concentration of the studied metals in the solutions after leaching in HCl medium (3 M) of the studied black masses, ratio S/L = 100 g/L, reaction time = 2 h, temperature = 70 °C. Error in all measures: ±0.1.

Metal	BM5 (g/L)	TUC2 (g/L)
Co	4.7	8.6
Ni	21.8	22.9
Mn	5.5	6.2
Cu	0.7	0.1
Li	0.6	1.8
IR *	58.9	46.6

* IR = insoluble residue.

**Table 15:** Extraction percentages of Cu(II) and Co(II) for leached black masses. Organic phase: Aliquat 336 0.6 M in kerosene with 10% decanol. Aqueous-to-organic ratio 1/1. Temperature 25 °C.

Black Mass	Stage	(Co)_AP_ * (g/L)	(Cu)_AP_ * (g/L)	E Co (%)	E Cu (%)
BM5	1	4.8 ± 0.1	0.2 ± 0.1	17.6 ± 0.1	72.9 ± 0.1
	2	4.0 ± 0.1	0.06 ± 0.02	24.4 ± 0.1	92.1 ± 0.1
	3	3.5 ± 0.1	0.01 ± 0.01	28.7 ± 0.1	97.9 ± 0.1
	4	2.7 ± 0.1	0.003 ± 0.002	45.3 ± 0.1	99.5 ± 0.1
TUC2	1	6.9 ± 0.1	0.03 ± 0.01	3.6 ± 0.1	79.3 ± 0.1
	2	6.2 ± 0.1	0.006 ± 0.004	12.9 ± 0.1	95.0 ± 0.1
	3	5.2 ± 0.1	0.002 ± 0.001	27.9 ± 0.1	98.7 ± 0.1
	4	3.9 ± 0.1	0.5 × 10^−3^ ± 0.0001	44.8 ± 0.1	99.6 ± 0.1

* AP = aqueous phase.

**Table 16:** Extraction percentages of Cu(II) and Co(II) for leached black masses. Organic phase: Aliquat 336 0.7 M in kerosene with 10% decanol. Aqueous-to-organic ratio 1/1. Temperature 25 °C.

Black Mass	Stage	(Co]_AP_ * (g/L)	(Cu)_AP_ * (g/L)	E Co (%)	E Cu (%)
BM5	1	4.0 ± 0.1	0.1 ± 0.1	15.4 ± 0.1	77.8 ± 0.1
	2	3.2 ± 0.1	0.03 ± 0.02	32.2 ± 0.1	95.4 ± 0.1
	3	2.3 ± 0.1	0.006 ± 0.004	50.5 ± 0.1	99.0 ± 0.1
	4	2.1 ± 0.1	0.001 ± 0.001	54.6 ± 0.1	99.8 ± 0.1
TUC2	1	5.5 ± 0.1	0.01 ± 0.01	36.9 ± 0.1	89.4 ± 0.1
	2	4.8 ± 0.1	0.003 ± 0.003	44.3 ± 0.1	99.5 ± 0.1
	3	4.2 ± 0.1	0.9 × 10^−3^ ± 0.0005	51.9 ± 0.1	99.9 ± 0.1
	4	3.5 ± 0.1	0.3 × 10^−3^ ± 0.0004	59.0 ± 0.1	100 ± 0.1

* AP = aqueous phase.

**Table 17:** Extraction percentages of Cu(II) and Co(II) for leached black masses under the most favorable extraction conditions. Organic phase: Aliquat 336 0.6 M in kerosene with 10% decanol. Aqueous-to-organic ratio 1/1. Temperature 25 °C.

Black Mass	Stage	(Co)_AP_ * (g/L)	(Cu)_AP_ * (g/L)	E Co (%)	E Cu (%)
BM5	1	4.0 ± 0.1	0.2 ± 0.1	5.1 ± 0.1	73.3 ± 0.1
	2	3.2 ± 0.1	0.05 ± 0.01	23.0 ± 0.1	91.8 ± 0.1
TUC2	1	6.9 ± 0.1	0.03 ± 0.01	12.2 ± 0.1	77.1 ± 0.1
	2	5.7 ± 0.1	0.007 ± 0.002	27.7 ± 0.1	93.8 ± 0.1

* AP = aqueous phase.

**Table 18:** Stripping percentages of Cu(II). Aqueous phase: H_2_SO_4_ 1 M. Aqueous-to-organic ratio 1/1. Temperature 25 °C.

Black Mass	Stage	(Cu)AP * (g/L)	RE Cu (%)
BM5	2	0.116 ± 0.001	95.8 ± 0.1
TUC2	2	0.019 ± 0.003	100 ± 0.1

* AP = aqueous phase.

**Table 19:** Extraction percentages of the metals contained in the studied samples.

Metal	BM5 (g/L)	TUC2 (g/L)	(BM5)_AP_ * (g/L)	(TUC2)_AP_ * (g/L)	E (BM5) (%)	E (TUC2) (%)
Co	0.55 ± 0.01	1.00 ± 0.001	0.038 ± 0.001	0.067 ± 0.006	93.0 ± 0.1	93.3 ± 0.1
Ni	4.87 ± 0.001	4.96 ± 0.001	4.96 ± 0.01	6.31 ± 0.01	0	0
Mn	1.04 ± 0.01	1.47 ± 0.001	0.68 ± 0.01	0.93 ± 0.01	34.4 ± 0.1	36.8 ± 0.1
Cu	0.009 ± 0.001	0.001 ± 0.001	0.003 ± 0.001	0.5 × 10^−3^ ± 0.0005	66.7 ± 0.1	50.0 ± 0.1
Li	0.11 ± 0.01	0.38 ± 0.01	0.14 ± 0.01	0.46 ± 0.0004	0	0

* AP = aqueous phase.

**Table 20:** Stripping percentages of Co(II) for studied samples.

Co_AP_ * (BM5) (g/L)	Co_AP_ * (TUC2) (g/L)	RE (BM5) (%)	RE (TUC2) (%)
0.42 ± 0.01	0.66 ± 0.01	82.5 ± 0.1	71.4 ± 0.1

* AP = aqueous phase.

## Data Availability

The original contributions presented in this study are included in the article/Supplementary Material. Further inquiries can be directed to the corresponding author(s).

## Supplementary Materials

The following supporting information can be downloaded at: https://www.mdpi.com/article/10.3390/molecules30224414/s1, Figure S1: Molecular structures of l-Menthol (left) and Aliquat 336 (right) with ptotons labeled (a-p) for NMR signal assignment; Table S1: Chemical shift values (δ, in ppm) of the labeled protons in L-Menthol-Aliquat 336 DES, after Co-extraction, and after Co-stripping.; Table S2: Weight percentage of the metals studied in the different black masses used.
