# Supplementary material for: Co(II) Recovery from Hydrochloric Acid Solution Using Menthol-Based Deep Eutectic Solvents (DESs): Application to NMC Battery Recycling
Source: Molecules. 2025 Nov 14;30(22):4414. doi: 10.3390/molecules30224414 (PMC12655439; doi:10.3390/molecules30224414)
Supplement: Supplementary file 1 [file molecules-30-04414-s001.zip › molecules-3912439-supplementary.pdf]

Supplementary information

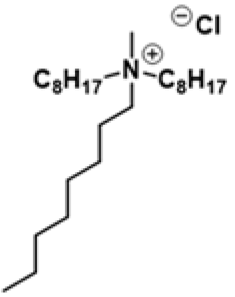

<u>Aliquat 336.</u> **^{1}H-RMN (500 MHz, DMSO-d6) δ (ppm)**: 4.86 (s, 3H, -N^+-$\boldsymbol{CH_3}$), 3.07 (m, 6H, -N^+-$\boldsymbol{CH_2}$-), 1.13 – 1.05 (m, 36H, -N^+-CH_2-$\boldsymbol{(CH_2)_n}$-CH_3); 0.71 (m, 9H, -$(CH_2)_n$-$\boldsymbol{CH_3}$).

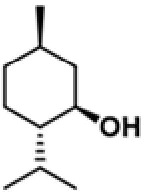

<u>L-Menthol.</u> **^{1}H-RMN (500 MHz, DMSO-d6) δ (ppm)** 2.87 (1H, C-H_g), 2.00 (1H, C-H_i); 1.73 (1H, C-H_d), 1.45 – 1.34 (4H, C-$H_{b,e}$), 1.13 – 1.05 (1H, C-H_c), 0.87 (1H, C-H_f); 0.71 (1H, C-H_d); 0.63 (3H, C-$H_{a,h}$), 0.55-0.53 (3H, C-H_j)

Figure S1. Assignment of the H of Aliquat 336 and of L-Menthol.

Table S1. Chemical composition of the studied black masses.

Metal	BM6 (wt%)	BM8 (wt%)	BM9 (wt%)	BM5 (wt%)	BM1 (wt%)	TUC2 (wt%)
Co	4.48	7.27	8.84	4.31	7.42	4.31
Ni	16.27	9.21	41.6	21.74	18.9	12.58
Mn	4.16	9.42	10.4	4.58	8.2	3.98
Cu	2.70	15.18	6.17	2.76	0.88	0.50
Li	3.37	2.30	0.8	0.62	0.85	1.21
